# Ssc-MiR-21-5p and Ssc-MiR-615 Regulates the Proliferation and Apoptosis of Leydig Cells by Targeting SOX5

**DOI:** 10.3390/cells11142253

**Published:** 2022-07-21

**Authors:** Qi Tang, Yanghai Zhang, Linxiu Yue, Hongying Ren, Chuanying Pan

**Affiliations:** 1 College of Animal Science and Technology, Northwest A&F University, Yangling, Xianyang 712100, China; tangqi777@nwafu.edu.cn (Q.T.); zhang2485@wisc.edu (Y.Z.); ylx2021@nwafu.edu.cn (L.Y.); renhongying90@nwsuaf.edu.cn (H.R.); 2 Department of Animal and Dairy Sciences, University of Wisconsin-Madison, Madison, WI 53706, USA

**Keywords:** ssc-miR-21-5p, ssc-miR-615, Leydig cells, proliferation, apoptosis, SOX5

## Abstract

Leydig cells (LCs) are the predominant cells of androgen production, which plays key roles in spermatogenesis and maintaining male secondary sexual characteristics. Abnormal development of LCs affects androgen levels in vivo, affects fertility and may even lead to infertility. Little is known about the regulation mechanism on LCs’ development and maturation in domestic animals, especially the regulation of non-coding RNAs. In this study, we continued to dig deeper in the previous RNA-seq data of porcine LCs from our group, combined with detecting the expression profiles in different tissues and different types of cells in the testis, to screen out candidate microRNAs (miRNAs) that may affect the regulation of LCs. A total of two miRNAs, ssc-miR-21-5p and ssc-miR-615 (“ssc” is omitted below), were finally determined. After overexpression and interference of miRNAs in vitro, the effects of candidate miRNAs on the proliferation and apoptosis of TM3 (mouse Leydig cell line) were explored. The results showed that miR-21-5p led to a decrease in TM3 cell density and p53 (apoptosis related protein) expression. Meanwhile, miR-21-5p decreased EdU positive cell numbers, but increased TUNEL positive cell numbers, suggesting miR-21-5p could inhibit proliferation and promote apoptosis. Conversely, miR-615 could increase TM3 cell density. Western blot and TUNEL assay indicated miR-615 inhibited apoptosis, but had no effect on proliferation. In addition, Sox5 was identified a potential target gene of these two miRNAs by Dual-Luciferase reporter system assay. Our findings about functions of miRNAs in TM3 and the mapping of miRNAs-target gene regulatory network would provide an important basis for the further elucidation of miRNAs in regulating pig LCs.

## 1. Introduction

MicroRNAs (miRNAs) are small regulatory RNAs that are processed from the stem-loop region of longer RNA transcripts and exert post-transcriptional inhibition by targeting mRNAs [1,2]. miRNAs exist in many eukaryotes and are highly conserved [3]. This RNA interference phenomenon regulates the expression of most mRNAs in humans, suggesting that miRNAs affect virtually all developmental processes and disease occurrence [4]. Especially, some miRNAs have participated in maintaining the normal function of male reproduction, including steroidogenesis, spermatogenesis, embryonic development and so on [5]. For instance, miR-150 plays a negative regulatory role in steroidogenesis of Leydig cells via targeting to STAR [6]. Testis-specific miR-469 silences TP2 and Prm2 in pachytene spermatocytes and round spermatids, which is critical to their timely translation at later times of spermiogenesis, finally attaining mature sperm [7]. By association analysis, miR-149 expressed in sperm is correlated with the quality of early embryonic development [8]. All these findings may provide theoretical support for the important role of miRNAs in male reproduction.

Leydig cells (LCs) are androgen producing cells located in the interstitium of testis, accounting for 2–4% of the volume of testis [9]. From the fetal stage, the development of LCs affects the normal physiological development of males. LCs not only promote the development and maturity of male reproductive organs and sperm, but also regulate the development of male secondary sexual characteristics through the secretion of androgens, for which their normal function is necessary for reproductive development [10]. At present, only a few studies exist about LCs in domestic animals. As an important pillar of the livestock industry in our country and an ideal mammalian model, pigs are still poorly understood about the regulation mechanism of LCs’ development and maturation, especially the regulation of non-coding RNAs. Therefore, studying the regulation mechanism of LCs is of great significance for the in-depth understanding of testicular development and disease treatment as well as the regulation of reproductive traits in domestic animals.

The development and maturation process of LCs is affected by hormones, cytokines and micro-environment. Currently, there are few reports on the regulation of the proliferation and apoptosis of pig LCs. Based on the pig stem Leydig cells (SLCs) isolation method and short-term culture system developed in our group, primary LCs (including SLCs and differentiated LCs) and ethane dimethane sulfonate (EDS)-treated LCs (the majority of cell type was SLCs) were successfully isolated for RNA-seq [11]. In this study, candidate miRNAs that might affect the regulation of LCs were selected by deeply mining RNA-seq data and their potential functions were explored via in vitro overexpression and interference tests. Subsequently, downstream target genes of candidate miRNAs were predicted and verified to explore regulatory patterns of miRNAs and target genes, which provide the theoretical foundation for revealing the regulation mechanism in pig LCs.

## 2. Material and Methods

The collection and preservation of the samples meet the relevant requirements of the Faculty Animal Policy and Welfare Committee of Northwest A&F University (protocol number NWAFAC1008).

### 2.1. Samples Collection

Tissue samples were collected from the adult (*n* = 3) Guanzhong black pigs, including heart, liver, spleen, kidney, testis, epididymis and brain. Then, they were wrapped in tinfoil paper and placed in liquid nitrogen, so as to be stored in a −80 °C freezer for subsequent RNA extraction.

### 2.2. Cell Culture and Transfection

TM3 (mouse Leydig cell line) (National Collection of Authenticated Cell Cultures (Serial: GNM24)), TM4 (mouse Sertoli cell line) (American Type Culture Collection, Shanghai, China (CRL-2053)) and GC1*_spg_* (mouse germ cell line) (American Type Culture Collection (CRL-1715)) were cultured in the incubator (5% CO_2_, 37 °C) using DMEM/high-glucose medium (BBI Biotech Co., Ltd., Shanghai, China), 10% fetal-bovine serum (Zeta Life Biotech Co., Ltd., Xi’an, Shannxi, China) and 1% penicillin/streptomycin (InCellGene LLC., Shanghai, China). The TM3 cells were seeded into 6-well plate with 2 × 10^5^ cells each well for subsequent treatment when reached 40%. The RNA oligo for transfection, including mimics, inhibitors of miRNAs and negative control (NC), inhibitor negative control (iNC) were synthesized (GenePharmaCo., Ltd., Shanghai, China). Using Lipofectamine 2000 (Invitrogen, Carlsbad, CA, USA), miR-21-5p (100 nM mimic/NC, 120 nM inhibitors/iNC) and miR-615 (50 nM mimic/NC, 100 nM inhibitors/iNC) were transfected according to the manufacturer’s recommendations. At 48 h post-transfection, the cells were collected and evaluated.

### 2.3. Quantitative Real-Time PCR

Total RNA from tissues or cells was isolated using TRIzol method, and its quality and integrity were detected using NanoDrop 2000 spectrophotometer (Thermo Scientific, Waltham, MA, USA) and 2% agarose gel electrophoresis. Then, the quantified RNA was reversely transcribed into cDNA using Hifair^®^III 1st Strand cDNA Synthesis Kit (Yeasen Biotech Co., Ltd., Shanghai, China). qRT-PCR was performed in a CFX96 Real-Time PCR System (Bio-Rad Laboratories, CA, USA) using SYBR qPCR Master Mix (Vazyme Biotech Co., Ltd., Nanjing, Jiangsu, China) and the primer sets. The primer sequences were listed in (Table 1). The expression levels of genes were normalized to β-actin, and that of miRNAs were normalized to U6 (2^−ΔΔCT^ analysis).

### 2.4. Protein Extraction and Western Blot

The cells of a 6-well plate were digested and collected into a centrifuge tube and subsequently lysed with RIPA (P0013B, Beyotime Biotech Co., Ltd., Shanghai, China) which contained 1 mmol/L PMSF (Beyotime Biotech Co., Ltd., Shanghai, China). A small amount of lysis products were used for BCA measurement, and the remaining split products were added into 5× loading buffers and bathed at 100 °C for 10 min. The denatured protein was stored at −80 °C.

According to the protocol described previously [11], the western blot was carried out using an antibody against BAX (CST, 1:1000), Bcl2 (1:500) (Proteintech Group, Inc., Chicago, IL, USA), β-Actin (Proteintech, 1:6000), p53 (1:1000) (Santa Cruz Biotechnology, Inc., Shanghai, China) and PCNA (Proteintech, 1:200). Protein grayscale analysis was conducted using ImageJ software (National Institutes of Health, Bethesda, MD, USA).

### 2.5. EdU Staining Assay

An EdU kit (BBI Biotech Co., Ltd., Shanghai, China) was used to detect the proliferation of cells. After incubated with medium containing 10 μM EdU for 2 h, the cells were processed via fixation, staining, permeation, etc. The nuclei were visualized using Hoechst 3342. Digital images were captured by fluorescence microscopy (Nikon Eclipse 80i, Tokyo, Japan). Cell counting was performed using ImageJ software.

### 2.6. TUNEL Staining Assay

One Step TUNEL Apoptosis Assay Kit (Beyotime Biotech Co., Ltd., Shanghai, China) was used to detect the apoptotic cells. The detecting method referred to the protocol provided by Beyotime. The nuclei were visualized using DAPI (BBI Biotech Co., Ltd., Shanghai, China). Digital images were captured by fluorescence microscopy. Cell counting was performed using ImageJ software.

### 2.7. Dual-Luciferase Reporter System Assay

Based on the results of target relationship prediction and sequence alignment, the 3′-UTR sequence of wild-type (WT) or mutation-type (Mut) within pig target genes binding with miRNAs’ seed region were designed, which were then cloned and ligated to the psi-Check2 Dual-Luciferase reporter vector by Tsingke Biotechnology Co., Ltd. (Beijing, China). According to the instructions, the obtained dry powder plasmids were diluted, transformed into Escherichia coli and extracted for the next transfection.

When the density of HeLa cells reached about 70% after being seeded into a 96-well plate with 7000 cells per well, 100 ng recombinant plasmids of WT or Mut were transfected into cells by Lipofectamine 2000 along with mimics or NC, respectively. Forty-eight hours after transfection, cells were washed with DPBS and lysed in a 100 μL prepared 1×Passive Lysis Buffer (Promega, Wisconsin, WI, USA) for 30 min. The lysate was collected into the centrifuge tube for high-speed centrifugation, and then 10 μL of supernatant was transferred to an opaque white microplate. The Firefly and Renilla luciferase activity was measured in turn using a multifunctional enzyme label instrument (BioTek Instruments, Inc., Winooski, VT, USA).

### 2.8. Statistical Analysis

In order to determine the differences in the mRNA, protein, cell numbers, etc., all data were analyzed using IBM SPSS Statistic 22. ANOVA followed by an LSD test was applied to compare differences in multiple groups, while differences between two groups were compared by Student’s t-test. The visualized results were displayed by GraphPad Prism software, and were expressed as the means ± standard errors (SE).

## 3. Results

**Figure 1:**
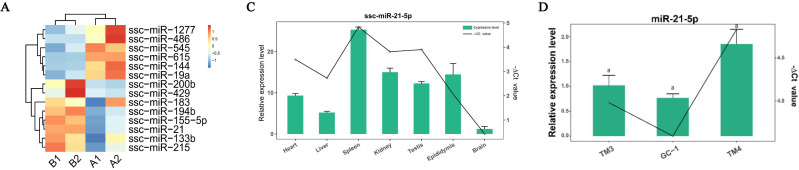

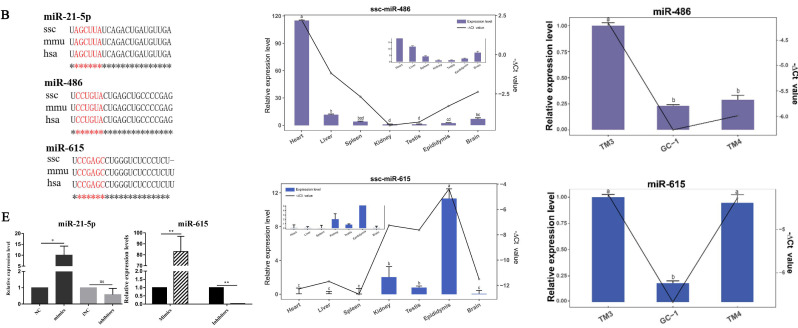
Preliminary screening and expression profile analysis of candidate miRNAs. (**A**) Cluster analysis between primary group (A1,A2) and EDS−treated group (B1,B2) [11]; (**B**) The alignment of three candidate miRNAs (miR-21-5p, miR-486 and miR-615) for pigs, mice, and humans. The red sequence represents the seed region; (**C**) Expression profiles of candidate miRNAs in different pig tissues; (**D**) Expression profiles of candidate miRNAs in mouse testis cells, including mouse Leydig cell line TM3, mouse Sertoli cell line TM4 and mouse germ cell line GC1*_spg_*; (**E**) miR-21-5p and miR-615 expression levels after overexpression or knockdown for 48 h. * Means *p* < 0.05, ** means *p* < 0.01. ns means no significant difference.

Preliminary screening and expression profile analysis of candidate miRNAs:

By analyzing the results of differentially expressed miRNAs [11], three candidate miRNAs, ssc-miR-21-5p, ssc-miR-486 and ssc-miR-615 (“ssc” is omitted below), were selected, of which miR-21-5p was highly expressed in the EDS-treated group, while miR-486 and miR-615 were highly expressed in the primary group (Figure 1A). To evaluate the similarity of miR-21-5p, miR-486 and miR-615 in different species, the mature sequences of miRNAs in pig, mouse and human were compared using the miRBase platform (https://www.mirbase.org/, accessed on 19 November 2021), and the similarity analysis indicated that these candidate miRNAs are all highly conserved, and whose seed regions are identical, implying functional conservation (Figure 1B). Subsequently, the expression profiles of these candidate miRNAs in various tissues of pigs were tested. The results showed that miR-21-5p was highly expressed in all tested tissues, especially in spleen tissue. miR-486 was highly expressed in pig heart tissue, even higher than the reference gene, but was expressed at a low level in other tissues, and miR-615 was highly expressed in epididymis compared with other tissues (Figure 1C). In addition, to further expand the regulatory network, candidate miRNAs expressions were detected in three main types of testis cells: TM3, TM4 and GC1*_spg_*. Among them, miR-21-5p was highly expressed in all cell lines, and miR-486 was relatively high-expressed in the TM3 cell line, and miR-615 was highly expressed in TM3 and TM4 cell lines (Figure 1D). Finally, combined with other research and experimental results, miR-21-5p and miR-615 were selected as target miRNAs for in-depth research (Figure 1E). Considering the low transfection efficiency of primary cells and the absence of pig Leydig cell lines, subsequent investigations were performed on mouse Leydig cell line TM3.

### 3.1. miR-21-5p Inhibits Proliferation and Promotes Apoptosis

**Figure 2:**
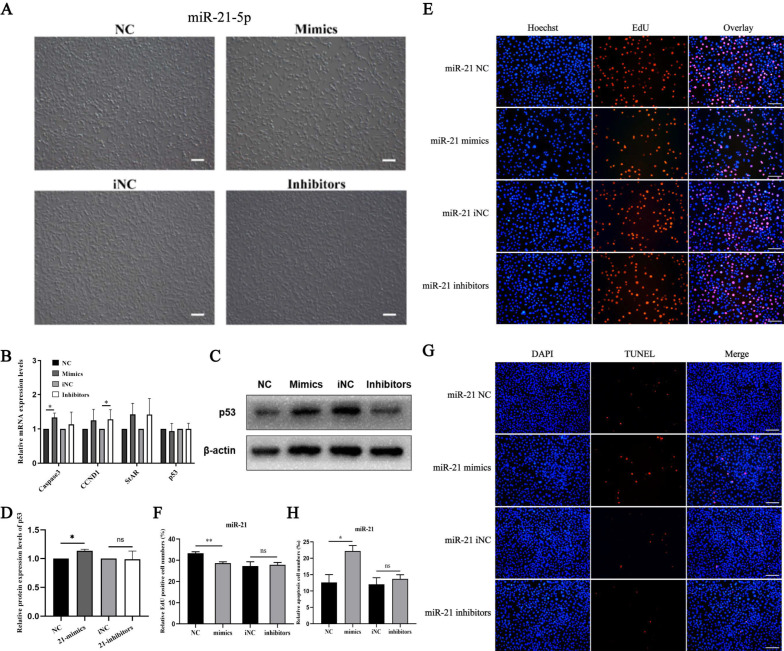
The effects of miR-21-5p on proliferation and apoptosis in TM3 cells. (**A**) The phenotypes of TM3 cells after over-expressing or inhibiting miR-21-5p; (**B**) Expression levels of several genes related to proliferation (CCND1), apoptosis (Caspase3, p53) and steroid synthesis (StAR) after miR-21-5p treatment for 48 h; (**C**,**D**) p53 protein expression level after overexpression or knockdown miR-21-5p in TM3 cells; (**E**,**F**) EdU assay was performed after miR-21-5p transfection for 48 h; (**G**,**H**) TUNEL assay was performed after miR-21-5p transfection for 48 h. Bar value is 100 μm. * Means *p* < 0.05, ** means *p* < 0.01, and ns means no significant difference.

In order to explore the function of miR-21-5p in TM3 cell proliferation or apoptosis, qRT-PCR, Western blot, EdU and TUNEL were performed. After miR-21-5p overexpressed for 48 h, the density of TM3 was significantly reduced, while inhibiting its expression, the density of cells did not change significantly (Figure 2A). Subsequently, the results of qRT-PCR showed miR-21-5p significantly increased mRNA expression levels of Caspase 3 (apoptosis-related marker) and StAR (steroid synthesis marker), but had no significant effects on CCND1 and p53 (Figure 2B). Western blot analysis revealed that the p53 expression level was decreased after miR-21-5p overexpression in TM3 cells (Figure 2C,D). The results of EdU experiments showed the percentage of positive EdU cells significantly reduced with up-regulation of miR-21-5p (Figure 2E,F). TUNEL assay showed miR-21-5p promoted cell apoptosis percentage (Figure 2G,H). These findings suggested that miR-21-5p may be involved in cell steroidogenesis, proliferation and apoptosis.

### 3.2. miR-615 Inhibits Apoptosis but Does Not Affect Proliferation

**Figure 3:**
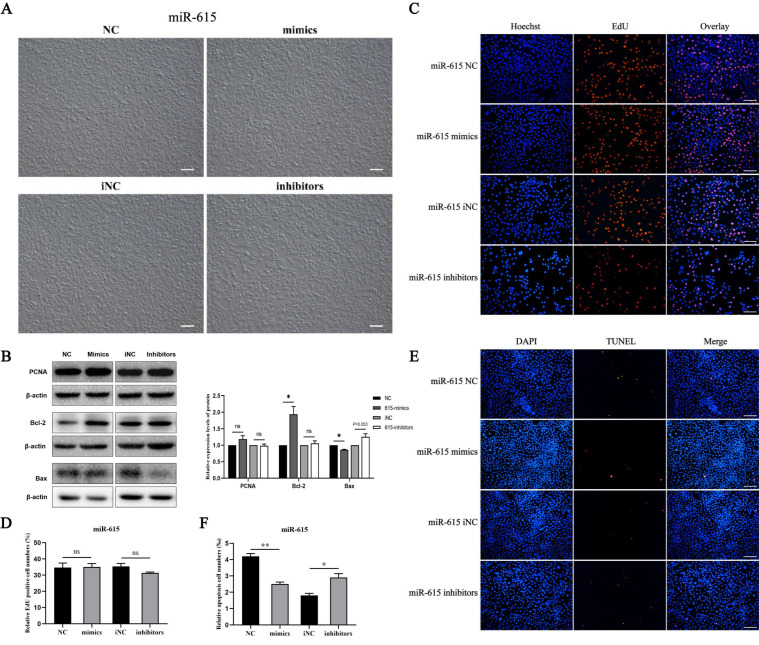
The effects of miR-615 on proliferation and apoptosis in TM3 cells. (**A**) The phenotypes of TM3 cells after over-expressing or inhibiting miR-615. (**B**) Proliferation (PCNA) and apoptosis (Bcl2, Bax)-related protein expression levels were detected after miR-615 treatment for 48 h. (**C**,**D**) EdU assay was performed after miR-615 transfection for 48 h. (**E**,**F**) TUNEL assay was performed after miR-615 transfection for 48 h. Bar value is 100 μm. * Means *p* < 0.05, ** means *p* < 0.01, and ns means no significant difference.

Cell phenotype showed that overexpression or knock-down miR-615 can increase or decrease cell numbers (Figure 3A). To investigate how miR-615 can affect TM3 cells, Western blotting was firstly performed and the results showed that Bcl-2 protein levels were significantly increased and Bax was significantly decreased in the mimic group. Compared with the iNC group, the expression of Bax was slightly increased when inhibiting miR-615 (Figure 3B). Meanwhile, TUNEL experiments indicated miR-615 can inhibit cell apoptosis according to the results of TUNEL positive cells with a significant reduction in the overexpression group and a significant increase in the knock-down group (Figure 3E,F). In addition, the PCNA expression level was not changed after miR-615 transfection (Figure 3B). EdU experiments also indicated miR-615 did not affect cell proliferation (Figure 3C,D). The aforementioned results demonstrated that miR-615 can inhibit the apoptosis of TM3 cells, but had no effect on proliferation.

### 3.3. The Targeting Relationship of Downstream Target Genes of miRNAs

**Figure 4:**
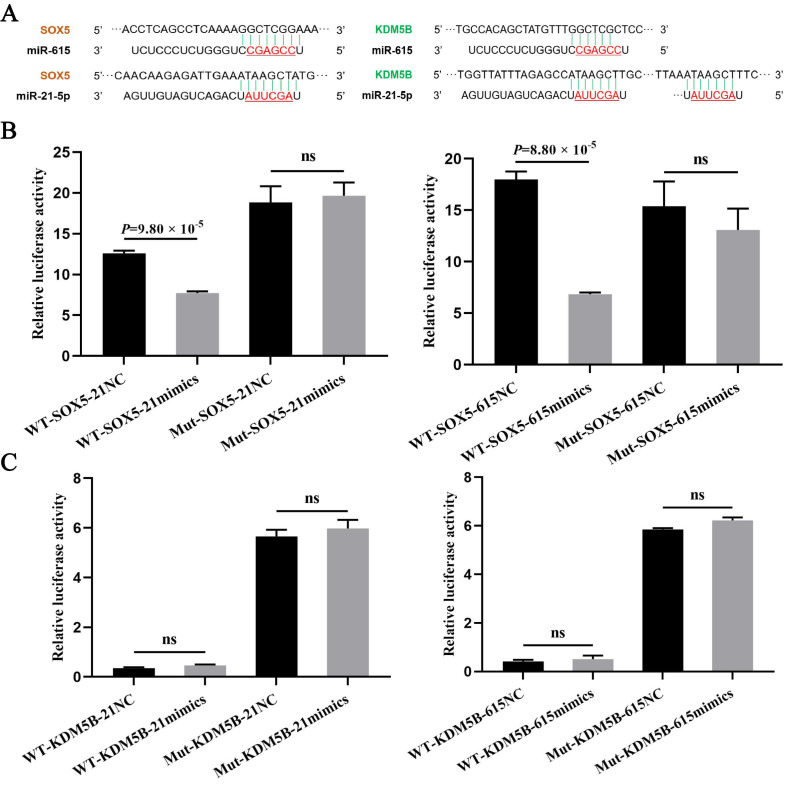
miR-21-5p and miR-615 can both target to SOX5 gene. (**A**) The schematic diagram of binding sites within miRNA and 3′ UTR of target genes (Red underlined means the seed region of miRNA). (**B**,**C**) The target relationship verification of two potential target genes (SOX5 and KDM5B) with miR-21-5p and miR-615, respectively, by Dual-Luciferase reporter assay. ns means no significant difference.

Using TargetScan and miRWalk 2.0 databases combined with sequence alignment, KDM5B and SOX5 were selected as candidate target genes of both miR-21-5p and miR-615 (Figure 4A). To verify their targeting relationship, the Dual-Luciferase report assays were performed after cotransfection for 48 h, and the results showed that the fluorescence activity of the SOX5-WT vector treated with miR-21-5p mimics was significantly lower than with NC. Meanwhile, the fluorescence of the SOX5-Mut vector after treatment with miR-205 or NC did not change significantly, indicating that SOX5 was a target gene of miR-21-5p (Figure 4B). Similarly, miR-615 can also target the SOX5 gene (Figure 4B). In addition, the fluorescence activity of the KDM5B-WT vector has no significant differences between miR-21-5p / miR-615 over-expressed groups and NC groups, which did not meet the expectations of targeting relationship (Figure 4C). Overall, miR-21-5p and miR-615 may target to SOX5 but not KDM5B for function regulation.

## 4. Discussion

Leydig cells are the main place of testosterone synthesis and secretion, and studying their proliferation and apoptosis has positive significance. As a member of non-coding RNAs, miRNAs play an important role in post-transcriptional regulation and are widely involved in the regulation of various biological processes. Based on the RNA-seq, a total of fifteen miRNAs were screened out with significantly different expression between the primary group and the EDS-treated group, among which miR-21-5p was highly expressed in the EDS-treated group; miR-486 and miR-615 were highly expressed in the primary group [11]. Among miR-486 and miR-615, the one that showed greater potential for male reproduction will be used for the preliminary study. To understand the function of miRNAs for next filtering, expression profiles in different tissues and different testicular germ cells were constructed through qRT-PCR, as miRNA is a small RNA with a length of 20–25 nt, and using qRT-PCR to detect its expression is reliable. The results showed miR-21-5p was wildly expressed in various tissues of pigs, which may have a wide range of effects. miR-486 was highly expressed in pig heart tissue but was expressed at a low level in other tissues, whereas miR-615 was highly expressed in epididymis and testis, suggesting a potential role in male reproduction. Meanwhile, three miRNAs were all highly expressed in the TM3 cell line. Lastly, miR-21-5p and miR-615 were predicted to target the same SOX5 gene. Briefly, all of the above have indicated that miR-21-5p and miR-615 may be involved in male reproduction. Thus, miR-21-5p and miR-615 were chosen for study.

miR-21 is one of the earliest miRNAs detected in the human genome, located within the intronic region of the TMEM49 gene. It is widely involved in many human diseases and cancer processes, and has received increased attention in recent years. A plethora of studies have found that miR-21 inhibits cell apoptosis and induces proliferation. Overexpressed miR-21 in mesenchymal stem cells can improve ovarian structure and function by inhibiting granulosa cells apoptosis, thereby repairing chemotherapy-induced premature ovarian failure [12]. Knockdown of ssc-miR-21-5p inhibited proliferation and migration of endometrial epithelial cells (EECs), and induced their apoptosis [13]. In spermatogonial stem cell (SSCs), transient inhibition of miR-21 increased the number of germ cells undergoing apoptosis, suggesting that it has a role in maintaining SSC self-renewal [14]. In contrast, Li et al. [15] showed that miR-21 exacerbates LPS-induced lung injury and modulates inflammatory response by exerting anti-proliferative and pro-apoptotic effects in normal human fibroblast cell. Similarly, we demonstrated that miR-21-5p can inhibit Leydig cells proliferation and induce their apoptosis.

miR-615 is located in the intron of humans or mice’s HOXC5 gene, and plays a pivotal role during cancer development and progression [16]. It is reported that miR-615 can promote cell proliferation and migration in breast cancer, stomach cancer, liver cancer, etc. However, miR-615 can also act as a tumor suppressor of lung cancer [17], esophageal cancer [18] and kidney cancer [19]. In addition, miR-615 serves as a potential therapeutic target of osteoarthritis [20], retinal neurodegeneration [21] and traumatic central nervous system injury [22]. Except for a few studies which speculated miR-615 may participate in the regulation of embryonic growth and gonadal differentiation, the involvement of miR-615 in reproduction processes is currently unknown [16]. In our research, we found that the miR-615-induced phenotype of increased Leydig cell number was the result of apoptosis inhibition, without relationship to proliferation.

**Figure 5:**
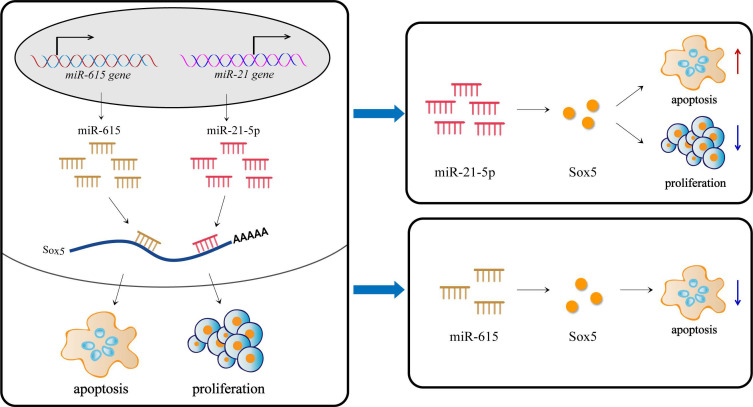
Proposed model of ssc-miR-21-5p and ssc-miR-615 regulation on Leydig cells proliferation and apoptosis by targeting SOX5.

miRNAs show differential expression and functions in various cells, and even act as both tumor suppressor genes and oncogenes, all of which may be explained in part by different target genes. We demonstrated using database prediction and sequence alignment, together with luciferase reporter assays, that SOX5 was a target gene of miR-21-5p and miR-615. The transcription factor encoded by SOX5 gene contains one or more highly conserved region of high mobility group which is with a specific DNA-binding domain and highly homologous to sex-determining genes. As a major postmeiotic transcriptional factor, plenty of studies have characterized the functions of SOX5 in testis development and spermatogenesis [23]. Zheng et al. [24] found the expression of SOX5 increases when round spermatids appear, indicating the important role at the spermatogenesis stage. In addition, the polymorphism of SOX5, which can lead to spermatogenic impairment, is associated with the risk of non-obstructive azoospermia [25,26,27]. Another research has revealed that SOX5, serving as a trans-acting factor, regulates male germ cell development in murine testes by stimulating miR-449 cluster expression [28]. Given these key functions of SOX5, we wondered that SOX5 might mediate the regulation process of miR-21-5p and miR-615 in Leydig cells proliferation and apoptosis (Figure 5). Notably, miR-21-5p and miR-615 targeted the same gene, but performed the opposite functions, which may regulate LCs both positively and negatively through complex mechanisms.

## 5. Conclusions

In Leydig cells, it was found that miR-21-5p could inhibit proliferation and promote apoptosis, while miR-615 could inhibit apoptosis. Meanwhile, it was proven that SOX5 was the target gene of two candidate miRNAs. These findings on the function of miRNAs in TM3 and the mapping of miRNAs-target gene regulatory network would provide an important basis for further elucidation of miRNAs in regulating pig LCs. However, the specific molecular mechanism on LCs’ development and maturation in domestic animals still needs further exploration.

## Figures and Tables

**Table 1:** The information of primers for RT-PCR and qRT-PCR.

Primer Name	Sequence (5′–3′)	Notes
miR-21-5p-slp	GTCGTATCCAGTGCAGGGTCCGAGGTATTCG CACTGGATACGACTCAACATC	stem-loop RT-PCR
miR-486-slp	GTCGTATCCAGTGCAGGGTCCGAGGTATTCG CACTGGATACGACCTCGGGGC	stem-loop RT-PCR
ssc-miR-615-slp	GTCGTATCCAGTGCAGGGTCCGAGGTATTCG CACTGGATACGACAGAGGGAG	stem-loop RT-PCR
mmu-miR-615-slp	GTCGTATCCAGTGCAGGGTCCGAGGTATTCG CACTGGATACGACAAGAGGGA	stem-loop RT-PCR
q-miR-21-5p-F	ATGGTTCGTGGGTAGCTTATCAGACTGA	qRT-PCR
q-miR-486-F	AACTGCAGAATCCTGTACTGAGCTG	qRT-PCR
q-miR-615-F	AAGGAAAAAATCCGAGCCTGGGTCTC	qRT-PCR
q-miR-U-R	GCAGGGTCCGAGGTATTC	Universal reverse primer of miRNAs for qRT-PCR
mus-U6	F: CGCTTCACGAATTTGCGTGTCAT	qRT-PCR
	R: GCTTCGGCAGCACATATACTAAAAT	
StAR	F: GGTTCTCAGCTGGAAGACACT	qRT-PCR (146 bp)
	R: ACCTCGTCCCCATTCTCCTG	
Caspase3	F: AGCTGGACTGTGGCATTGAG	qRT-PCR (143 bp)
	R: CCACGACCCGTCCTTTGAAT	
CCND1	F: CATTCCCTTGACTGCCGAGA	qRT-PCR (177 bp)
	R: TTGTTCTCATCCGCCTCTGG	
p53	F: ATGCGGTTCGGGTCCAAAAT	qRT-PCR (154 bp)
	R: CTAAATGGCAGTCGTTCTCTCC	
β-actin	F: CTCCATCATGAAGTGCGACGT	qRT-PCR (114 bp)
	R: GTGATCTCCTTCTGCATCCTGTC	

Note: The “ssc” means pig source, “mus” means mice source and “unspecified” means universal.

## Data Availability

The data presented in this study are available in the article.
